# Author Correction: Covalent coupling of Spike’s receptor binding domain to a multimeric carrier produces a high immune response against SARS-CoV-2

**DOI:** 10.1038/s41598-022-05984-4

**Published:** 2022-01-25

**Authors:** Argentinian AntiCovid Consortium, Paula M. Berguer, Matías Blaustein, Luis M. Bredeston, Patricio O. Craig, Cecilia D’Alessio, Fernanda Elias, Paola C. Farré, Natalia B. Fernández, Hernán G. Gentili, Yamila B. Gándola, Javier Gasulla, Gustavo E. Gudesblat, María G. Herrera, Lorena I. Ibañez, Tommy Idrovo-Hidalgo, Alejandro D. Nadra, Diego G. Noseda, Carlos H. Paván, María F. Pavan, María F. Pignataro, Ernesto A. Roman, Lucas A. M. Ruberto, Natalia Rubinstein, María V. Sanchez, Javier Santos, Diana E. Wetzler, Alicia M. Zelada

**Affiliations:** 1 Buenos Aires, Argentina; 2 Consejo Nacional de Investigaciones Científicas y Técnicas, Godoy Cruz 2290, C1425FQB Buenos Aires, Argentina; 3 Fundación Instituto Leloir, IIBBA, Consejo Nacional de Investigaciones Científicas y Técnicas (CONICET), Buenos Aires, Argentina; 4 Facultad de Ciencias Exactas y Naturales, Instituto de Biociencias, Biotecnología y Biología Traslacional (iB3), Universidad de Buenos Aires, Intendente Güiraldes 2160, Ciudad Universitaria, C1428EGA Buenos Aires, Argentina; 5 Departamento de Fisiología y Biología Molecular y Celular, Universidad de Buenos Aires Facultad de Ciencias Exactas y Naturales-Universidad de Buenos Aires, Intendente Güiraldes 2160, Ciudad Universitaria, C1428EGA Buenos Aires, Argentina; 6 Instituto de Química y Fisicoquímica Biológicas, Facultad de Farmacia y Bioquímica, Universidad de Buenos Aires, Junín 956, 1113AAD Buenos Aires, Argentina; 7 Departamento de Química Biológica, Facultad de Farmacia y Bioquímica, Universidad de Buenos Aires, Buenos Aires, Argentina; 8 Departamento de Química Biológica, Facultad de Ciencias Exactas y Naturales, Universidad de Buenos Aires, Intendente Güiraldes 2160, Ciudad Universitaria, C1428EGA Buenos Aires, Argentina; 9 Instituto de Química Biológica de la Facultad de Ciencias Exactas y Naturales (IQUIBICEN-CONICET), Buenos Aires, Argentina; 10 Instituto de Ciencia y Tecnología Dr. César Milstein (Consejo Nacional de Investigaciones Científicas y Técnicas-Fundación Pablo Cassará), Saladillo 2468, C1440FFX Buenos Aires, Argentina; 11 Laboratorio Pablo Cassará S.R.L., Buenos Aires, Argentina; 12 Centro de Investigaciones del Medio Ambiente (UNLP-CONICET), La Plata, Buenos Aires Argentina; 13 Departamento de Química Inorgánica, Analítica y Química Física, Facultad de Ciencias Exactas y Naturales, Universidad de Buenos Aires, Instituto de Química Física de los Materiales, Medio Ambiente y Energía (INQUIMAE CONICET), C1428EGA Buenos Aires, Argentina; 14 Universidad Nacional de San Martín-CONICET, Instituto de Investigaciones Biotecnológicas (IIBio), San Martín, Buenos Aires Argentina; 15 Instituto de Química y Fisicoquímica Biológicas, LANAIS PROEM, Facultad de Farmacia y Bioquímica, Universidad de Buenos Aires, Junín 956, 1113AAD Buenos Aires, Argentina; 16 Departamento de Microbiología, Inmunología, Biotecnología y Genética, Facultad de Farmacia y Bioquímica, Universidad de Buenos Aires, Buenos Aires, Argentina; 17 CONICET-Universidad de Buenos Aires, Facultad de Farmacia y Bioquímica, Instituto de Nanobiotecnología (NANOBIOTEC), Buenos Aires, Argentina; 18 Instituto Antártico Argentino, Ministerio de Relaciones Exteriores y Culto, Buenos Aires, Argentina; 19 Instituto de Medicina y Biología Experimental de Cuyo (IMBECU), Centro Científico Tecnológico de Mendoza (CCT-Mendoza), CONICET, Universidad Nacional de Cuyo, 5500 Mendoza, Argentina; 20 Facultad de Ciencias Exactas y Naturales, Instituto de Biodiversidad y Biología Experimental y Aplicada (IBBEA-UBA-CONICET), Buenos Aires, Argentina

Correction to: *Scientific Reports* 10.1038/s41598-021-03675-0, published online 13 January 2022

The original version of this Article contained an error in Figure 7 where a preliminary rendition was published. The original Figure 7 and accompanying legend appear below.

**Figure 7:**
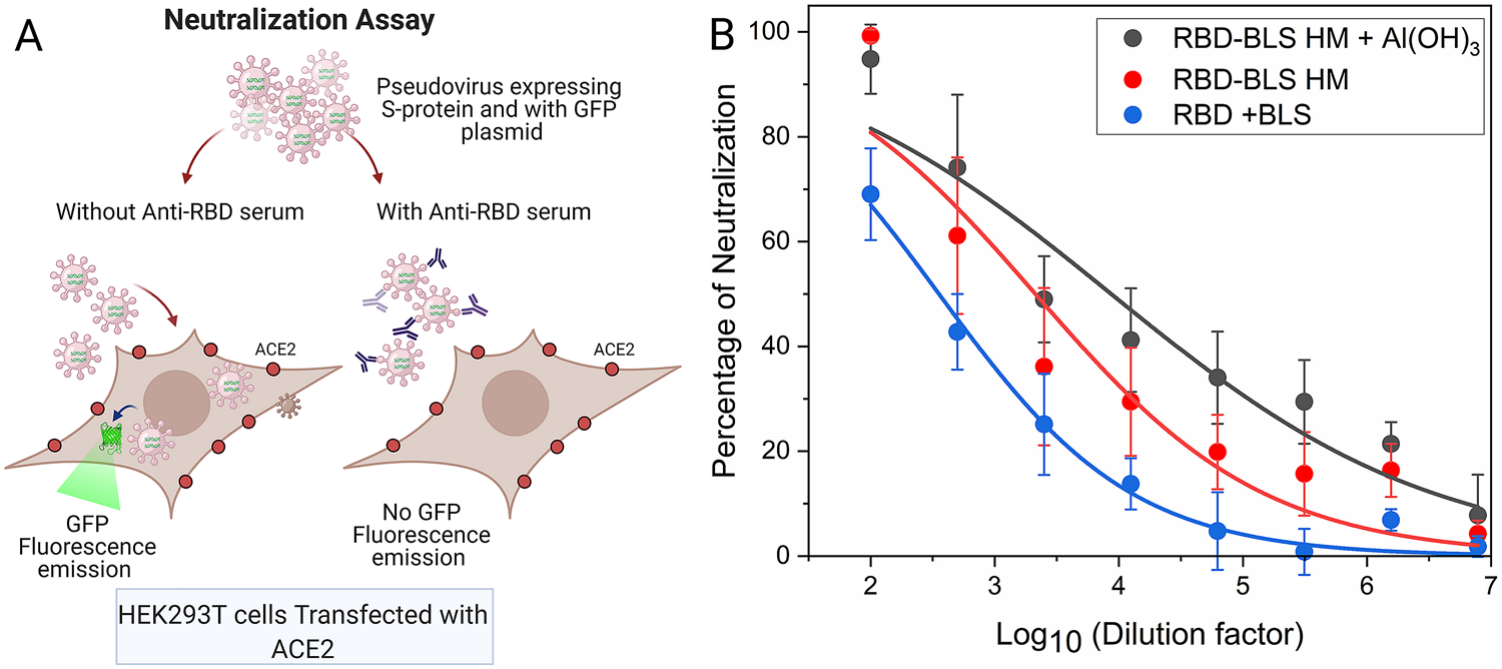
SARS-CoV-2 S pseudotyped virus neutralization assay. (**A**) Scheme of the assay. HEK-293T cells transfected with ACE2 and TMPRSS2 protease were transduced with a SARS-CoV-2 S pseudotyped lentivirus carrying a GFP-encoding mRNA in the presence of different dilutions of mouse sera. Forty eight hours later, cells were observed under the microscope. Created with BioRender.com. (**B**) The number of GFP positive cells for each serial dilution was determined. The serum antibody dilution causing a 50% reduction of GFP positive cells (IC_50_) compared to control “virus only” treated cells was calculated using Graphpad Prism. PreIS corresponds to the preimmune sera.

The original Article has been corrected.

