# Supplementary material for: Covalent coupling of Spike’s receptor binding domain to a multimeric carrier produces a high immune response against SARS-CoV-2
Source: Sci Rep. 2022 Jan 13;12:692. doi: 10.1038/s41598-021-03675-0 (PMC8758758; doi:10.1038/s41598-021-03675-0)
Supplement: Supplementary file 1 — Supplementary Information. [file 41598_2021_3675_MOESM1_ESM.pdf]

Supplementary Material

Covalent coupling of Spike's Receptor Binding Domain to a Multimeric Carrier produces a high immune response against SARS-CoV-2

Argentinian AntiCovid Consortium †

† Argentinian AntiCovid Consortium*: Correspondence and requests for materials should be addressed to the Argentinian AntiCovid Consortium (email: anticovid.arg@gmail.com). A comprehensive list of consortium members appears below. All authors (listed in alphabetical order) contributed equally to this work.

Paula M. Berguer[1,2,*], Matías Blaustein[1,3,4,*], Luis M. Bredeston[5,6,*], Patricio O. Craig[7,8,*], Cecilia D'Alessio[1,3,4,*], Fernanda Elias[9,*], Paola C. Farré[10], Natalia B. Fernández[1,3,4,*], Hernán G. Gentili[3,4,*], Yamila B. Gándola[3,4,*], Javier Gasulla[3,4,11,*], Gustavo E. Gudesblat[1,3,4,*], María G. Herrera[1,3,4,*], Lorena I. Ibañez[1,12,*], Tommy Idrovo-Hidalgo[3,4,*], Alejandro D. Nadra[1,3,4,*], Diego G. Noseda[13,*], Carlos H. Paván[1,14,*], María F. Pavan[1,12,*] María F. Pignataro[3,4,*], Ernesto A. Roman[5,7,*], Lucas A. M. Ruberto[15,16,17,*], Natalia Rubinstein[1,3,4,*] María V. Sanchez[18,*], Javier Santos[1,3,4,7,*], Diana E. Wetzler[7,8,*], Alicia M. Zelada[4,19,*]

[1]Consejo Nacional de Investigaciones Científicas y Técnicas. Godoy Cruz 2290 C1425FQB, Buenos Aires, Argentina.

[2]Fundación Instituto Leloir, IIBBA, Consejo Nacional de Investigaciones Científicas y Técnicas (CONICET), Buenos Aires, Argentina.

[3]Universidad de Buenos Aires, Facultad de Ciencias Exactas y Naturales, Instituto de Biociencias, Biotecnología y Biología Traslacional (iB3). Intendente Güiraldes 2160, Ciudad Universitaria, C1428EGA, Buenos Aires, Argentina.

[4]Departamento de Fisiología y Biología Molecular y Celular. Universidad de Buenos Aires Facultad de Ciencias Exactas y Naturales- Universidad de Buenos Aires, Argentina. Intendente Güiraldes 2160, Ciudad Universitaria, C1428EGA, Buenos Aires, Argentina.

[5]Instituto de Química y Fisicoquímica Biológicas. Facultad de Farmacia y Bioquímica, Universidad de Buenos Aires, Junín 956, 1113AAD, Buenos Aires, Argentina.

[6]Departamento de Química Biológica , Facultad de Farmacia y Bioquímica, Universidad de Buenos Aires, Buenos Aires, Argentina.

[7]Departamento de Química Biológica. Facultad de Ciencias Exactas y Naturales. Universidad de Buenos Aires. Intendente Güiraldes 2160, Ciudad Universitaria, C1428EGA, Buenos Aires, Argentina.

[8]Instituto de Química Biológica de la Facultad de Ciencias Exactas y Naturales (IQUIBICEN-CONICET). Buenos Aires, Argentina.

[9]Instituto de Ciencia y Tecnología Dr. César Milstein (Consejo Nacional de Investigaciones Científicas y Técnicas-Fundación Pablo Cassará), Saladillo 2468, C1440FFX Buenos Aires, Argentina.

[10]Laboratorio Pablo Cassará S.R.L.

[11]Centro de Investigaciones del Medio Ambiente (UNLP-CONICET), La Plata, Buenos Aires, Argentina

[12]Departamento de Química Inorgánica, Analítica y Química Física, Facultad de Ciencias Exactas y Naturales, Universidad de Buenos Aires. Instituto de Química Física de los Materiales, Medio Ambiente y Energía (INQUIMAE CONICET), C1428EGA, Buenos Aires, Argentina

[13]Universidad Nacional de San Martín-CONICET, Instituto de Investigaciones Biotecnológicas (IIBio), San Martín, Buenos Aires, Argentina

[14]Instituto de Química y Fisicoquímica Biológicas, LANAIS PROEM, Facultad de Farmacia y Bioquímica, Universidad de Buenos Aires, Junín 956, 1113AAD, Buenos Aires, Argentina.

[15]Departamento de Microbiología, Inmunología, Biotecnología y Genética, Facultad de Farmacia y Bioquímica, Universidad de Buenos Aires, Buenos Aires, Argentina.

[16]CONICET-Universidad de Buenos Aires, Facultad de Farmacia y Bioquímica, Instituto de Nanobiotecnología (NANOBIOTEC), Buenos Aires, Argentina.

[17]Instituto Antártico Argentino, Ministerio de Relaciones Exteriores y Culto, Buenos Aires, Argentina.

[18]Instituto de Medicina y Biología Experimental de Cuyo (IMBECU).Centro Científico Tecnológico de Mendoza (CCT-Mendoza), CONICET, Universidad Nacional de Cuyo, (5500), Mendoza, Argentina.

[19]Instituto de Biodiversidad y Biología Experimental y Aplicada (IBBEA-UBA-CONICET). Facultad de Ciencias Exactas y Naturales. Buenos Aires, Argentina.

Sequences of proteins used in this work.

Sortase A pentamutant (eSrtA):

MQAKPQIPKDKSKVAGYIEIPDADIKEPVYPGPATREQLNRGVSFAEENESLDDQNISIAG
HTFIDRPNYQFTNLKAAKKGSMVYFKVGNETRKYKMTSIRNVKPTAVEVLDEQKGKDK
QLTLITCDDYNEETGVWETRKIFVATEVKLEHHHHHH

(yellow, C-terminal Histag)

BLS sequence (modified for coupling by Sortase A enzyme):

MHMENLYFQGGGGSGSGLKTSFKIAFIQARWHADIVDEARKSFVAELAAKTGGSVEVEI
FDVPGAYEIPLHAKTLARTGRYAAIVGAAFVIDGGIYRHDFVATAVINGMMQVQLETEVP
VLSVVLTPHHFHESKEHHDFFHAHFKVKGVEAAHAALQIVSERSRIAALV

(in green, the TEV site and in orange a Sortase A site (N-terminal Gly-Gly-Gly) and a short linker Ser-Gly-Ser-Gly)

ACE2 Soluble receptor domain sequence:

MSSSSWLLLSLVAVTAAQSTIEEQAKTFLDKFNHEAEDLFYQSSLASWNYNTNITEENVQ
NMNNAGDKWSAFLKEQSTLAQMYPLQEIQNLTVKLQLQALQQNGSSVLSEDKSKRLN
TILNTMSTIYSTGKVCNPDNPQECLLLEPGLNEIMANSLDYNERLWAWESWRSEVGKQL
RPLYEEYVVLKNEMARANHYEDYGDYWRGDYEVNGVDGYDYSRGQLIEDVEHTFEEI
KPLYEHLHAYVRAKLMNAYPSYISPIGCLPAHLLGDMWGRFWTNLYSLTVPFGQKPNID
VTDAMVDQAWDAQRIFKEAEKFFVSVGLPNMTQGFWENSMLTDPGNVQKAVCHPTAW
DLGKGDFRILMCTKVTMDDFLTAHHEMGHIQYDMAYAAQPFLLRNGANEGFHEAVGEI
MSLSAATPKHLKSIGLLSPDFQEDNETEINFLLKQALTIVGTLPFTYMLEKWRWMVFKGE
IPKDQWMKKWWEMKREIVGVVEPVPHDETYCDPASLFHVSNDYSFIRYYTRTLYQFQF
QEALCQAAKHEGPLHKCDISNSTEAGQKLFNMLRLGKSEPWTLALENVVGAKNMNVR
PLLNYFEPLFTWLKDQNKNSFVGWSTDWSPYADGSGHHHHHHHH

(green, the natural signal peptide of ACE2 and in yellow, a short linker Gly-Ser-Gly and the C-terminal His tag)

RBD sequence for *Pichia pastoris* expression:

MRFPSIFTAVLFAASSALAAPVNTTTEDETAQIPAEAVIGYSDLEGDFDVAVLPFSNSTNNG
LLFINTTIASIAAKEEGVSLEKREAEAEFRVQPTESIVRFPNITNLCPFGEVFNATRFASVYA
WNRKRISNCVADYSVLYNSASFSTFKCYGVSPTKLNDLCFTNVYADSFVIRGDEVRQIAP
GQTGKIADYNYKLPDDFTGCVIAWNSNNLDSKVGGNYNYLYRLFRKSNLKPFERDISTE
IYQAGSTPCNGVEGFNCYFPLQSYGFQPTNGVGYQPYRVVVLSFELLHAPATVCGPKKS
TNLVKNKLPETGHHHHHH

(green alpha factor, yellow Sortase A and C-terminal Histag sequences)

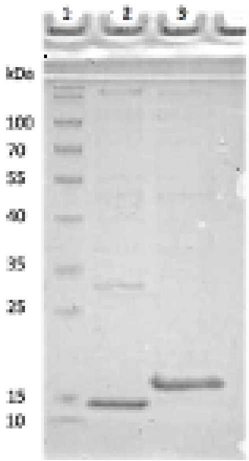

Figure S1. SDS-PAGE Analysis of BLS Digestion by TEV Protease. Lane 1: molecular weight markers, lane 2: BLS treated with TEV protease and lane 3: intact BLS. BLS was purified by ion exchange chromatography followed by SEC-HPLC**.**

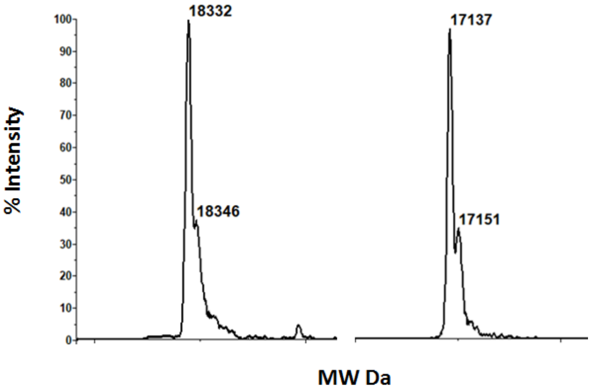

Figure S2. ESI-MS Spectra of BLS. Spectra corresponding to *Met-Glu-Asn-Leu-Tyr-Phe-Gln*-Gly-Gly-Gly-**BLS** (left) and Gly-Gly-GLy-**BLS**, after digestion with TEV protease (right) are shown.

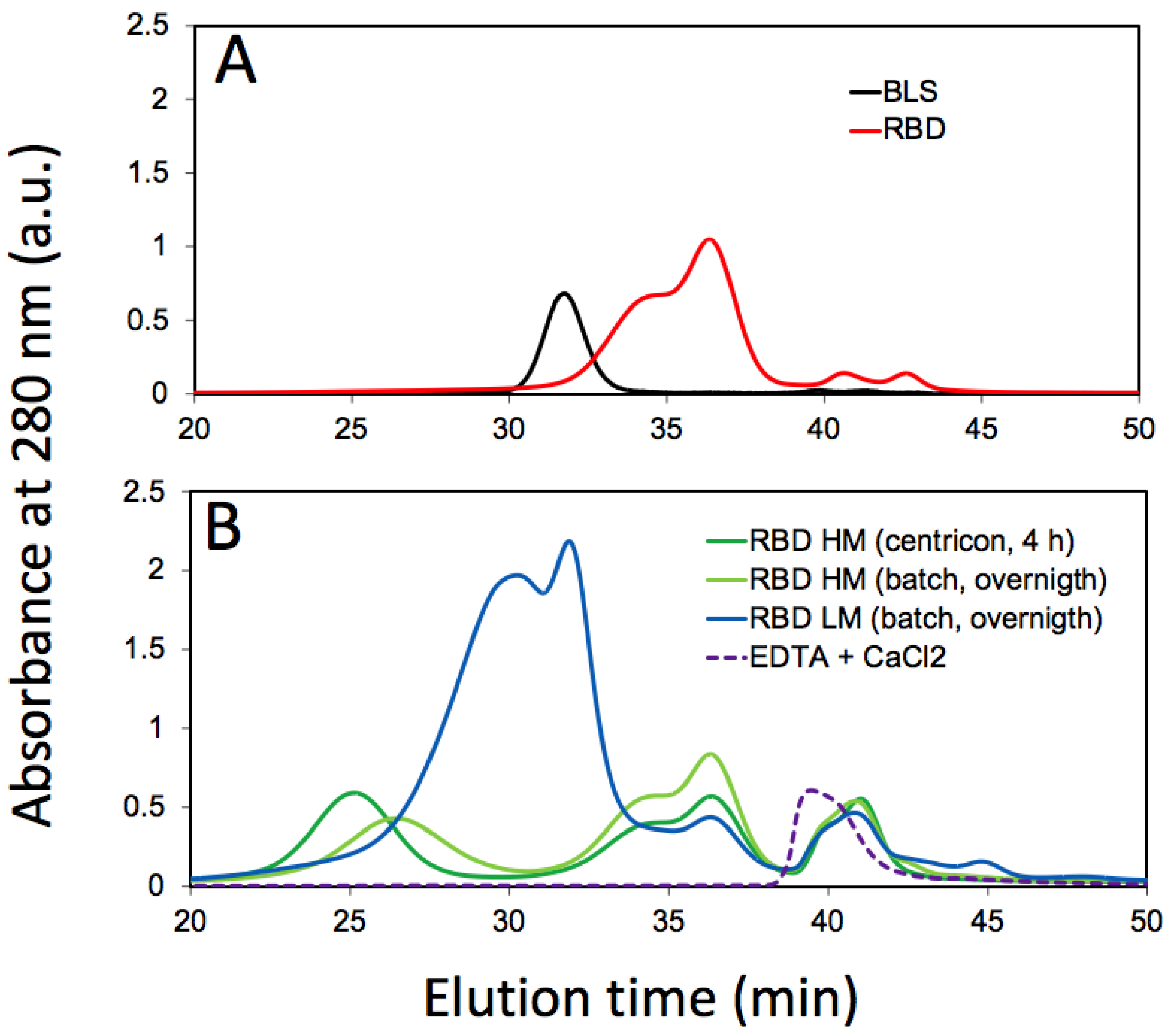

Figure. S3. Sortase A-mediated covalent coupling of RBD and BLS at low and high multiplicities. SEC-HPLC profiles corresponding to (A) Gly-Gly-Gly-BLS (black), RBD (red) and (B) transpeptidation reaction mixtures catalyzed by Sortase A under conditions for high multiplicity products (centricon 4h, dark green, and batch overnight, light green) and for low multiplicity products (blue). An equivalent volume of a 10 mM EDTA, 10 mM $CaCl_2$ solution was loaded as a control (magenta, lower pane). Decameric BLS has a molecular weight of approximately 170 kDa and each RBD subunit adds approximately 26 kDa or 40 kDa (excluding or including glycosylation, respectively). The peak. observed between 38-43 min corresponds to EDTA-Ca^{2+}.

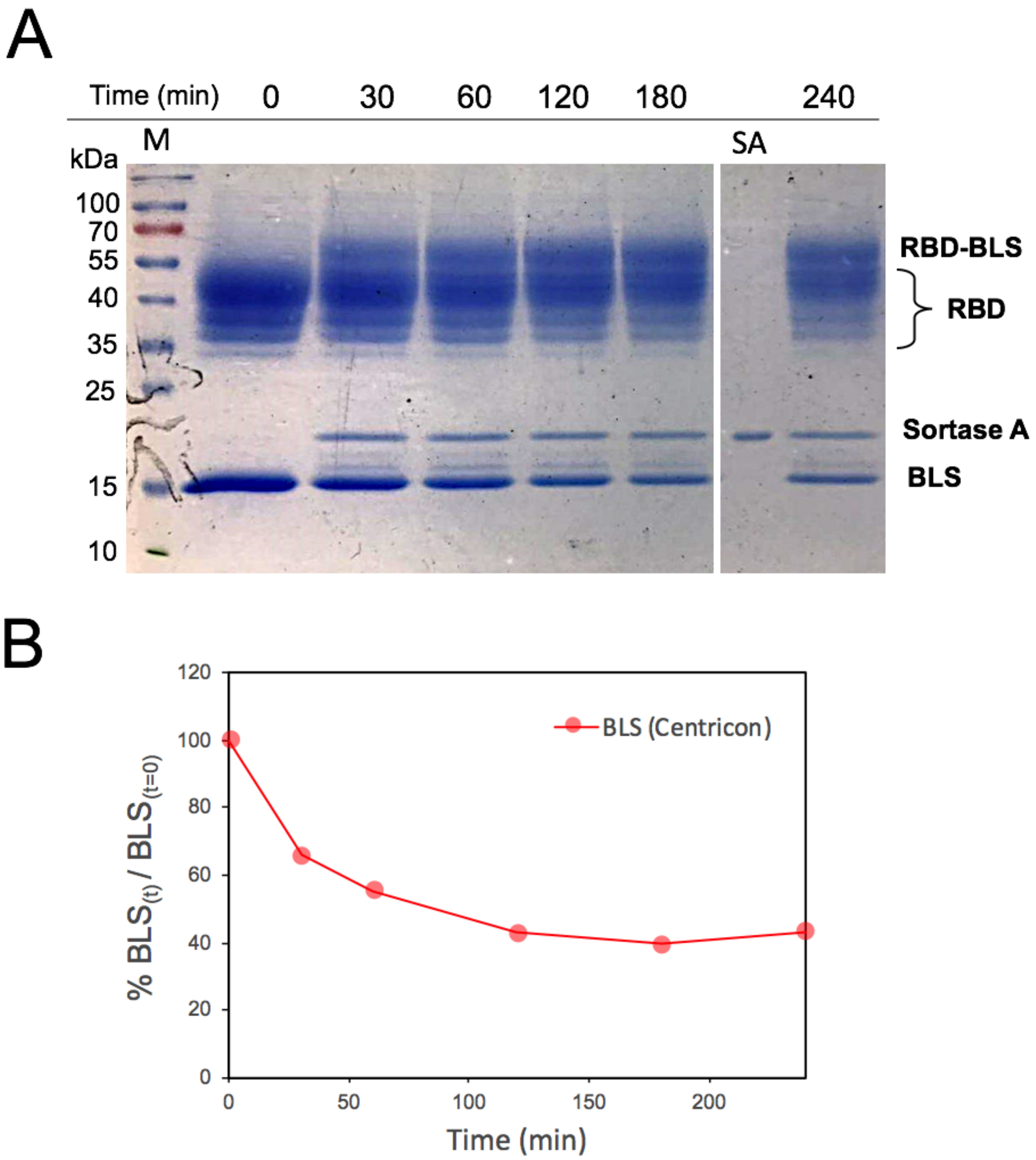

Figure S4. Monitoring RBD-BLS Covalent Coupling. (A) Sortase A reaction was monitored by an SDS-PAGE analysis. The reaction was carried out at 4 ºC in a centrifugal filter unit (Centricon), as described in Materials and Methods. Small samples were separated at reaction times of 0, 30, 60, 120, 180 and 240 min. Sortase A (SA) was included as a control, and molecular weight markers (M) were included as a reference. (B) The quantification of the loss of the BLS band in SDS-PAGE. The ratio $BLS_{(t)}$ /$BLS_{(t=0)}$ was calculated for each reaction time and was plotted as a percentage. $BLS_{(t)}$ and $BLS_{(t=0)}$ are the initial and the remaining BLS masses estimated from the analysis of the band density corresponding to the BLS band.

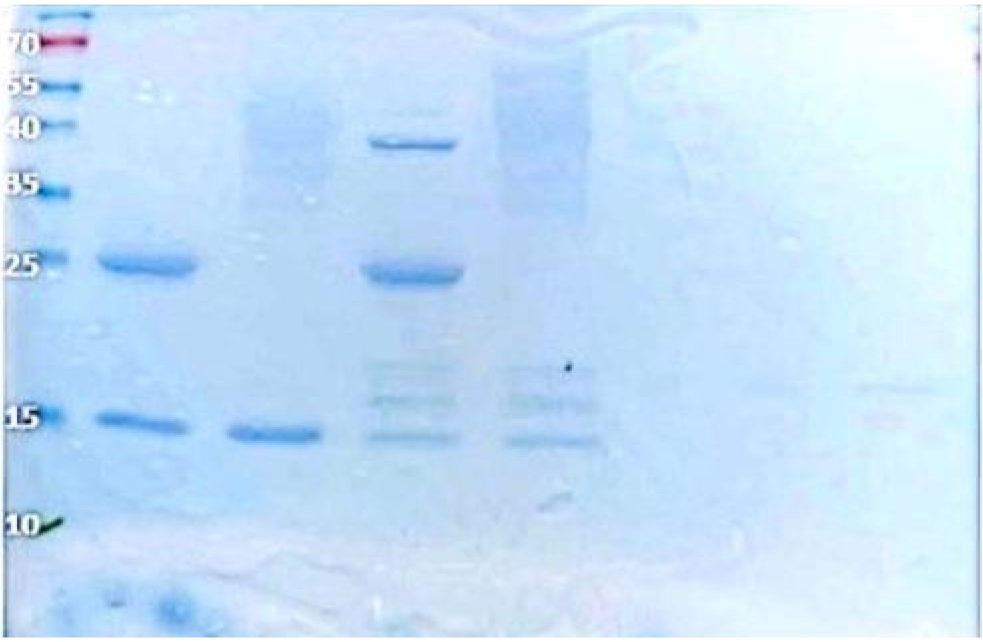

Figure S5. Full Image of the SDS-PAGE Presented in Figure 5 (Sortase A-mediated Covalent Coupling of RBD and BLS, main text).
